# A maturity matrix and actionable tool for implementing best practices within the radiography support workforce: a mixed methods synthesis

**DOI:** 10.1186/s12913-025-13888-y

**Published:** 2025-12-11

**Authors:** Sally Fowler-Davis, Julie Nightingale, Beverly Snaith, Sarah Etty, Trudy Sevens

**Affiliations:** 1 Faculty of Health Medicine and Social Care, Anglia Ruskin University, Cambridge, UK; 2 School of Health and Social Care, Centre for Applied Health and Social Care Research, Sheffield Hallam University, Sheffield, UK; 3 Faculty of Health Studies, University of Bradford, Bradford, UK; 4 Mid Yorkshire Teaching NHS Trust, Wakefield, UK

**Keywords:** Assistant practitioner, Deployment, Imaging, Radiographer, Radiology, Staffing, Support worker, Workforce

## Abstract

**Background:**

Radiology is a multidisciplinary specialty, combining the medical specialism of radiologists with the clinical expertise of diagnostic radiographers. Radiographers are skilled in performing imaging procedures such as X-Rays, ultrasound and computed tomography scans to diagnose and monitor conditions within a wide range of patient pathways, and they are assisted by support workers who enable service delivery by providing patient facing and ancillary activities. Imaging service demands in the United Kingdom continue to outpace growth in the radiographer workforce, and there is an urgent need to explore the potential for developing the capability and capacity of the imaging support workforce. A multi-centre mixed methods study investigated the determinants for the utilisation of the radiography support workforce in England, presenting the findings in a maturity matrix. A maturity matrix is an actionable tool which aims to facilitate practice improvements, presented as a series of distinct, iterative steps that showcase the desired developmental path towards an effective service.

**Methods:**

The mixed methods study employed an explanatory sequential, multi-stage advanced framework design, involving six consecutive workstreams. The final workstream synthesised and integrated findings from the previous workstreams to identify the key factors that contribute to best practices in imaging support workforce deployment. Documentation of critical determinants and articulation of quality indicators were modelled into a maturity matrix to be used by imaging managers to review and plan the development of their support workforce.

**Results:**

The Imaging Support Workforce Maturity Matrix is constructed as a means of tackling workforce improvements and tracking progress over time at service level. Fifteen critical determinants within three themed categories (evidence-based workforce planning; deployment; development and progression) were embedded within the matrix. Each determinant is presented for self-assessment against four levels of service maturity (Emerging, Developing, Maturing, and Thriving). These support assessment and action-planning towards the goal of fully developing the role and progression route for the support workforce.

**Conclusion:**

The Imaging Support Workforce Maturity Matrix is presented. The actionable tool was reviewed with service managers in a first stage of validation and further research will be undertaken to implement appropriately across other allied health professions.

**Supplementary Information:**

The online version contains supplementary material available at 10.1186/s12913-025-13888-y.

## Background

Medical imaging is a multidisciplinary specialty, combining the medical specialism of radiology with the clinical expertise of diagnostic radiographers, a profession recognised as having persistent shortages in healthcare systems worldwide [1]. In the United Kingdom, imaging services within the National Health Service (NHS) are underpinned by support workers and assistant practitioners (SWAPs), collectively referred to as the imaging support workforce [2, 3]. This non-registered workforce occupies bands 2–4 on the NHS Terms and Conditions of Service (Agenda for Change) banding system; registered practitioners span bands 5 to 6, while advanced and consultant practitioners normally occupy bands 7 and 8 respectively [4]. Effective utilisation of the support workforce is essential to counter a well-documented imaging workforce crisis resulting from a long-standing imbalance between workforce supply and clinical demand [1, 3].

Deploying the right number of staff with the appropriate skills and qualifications is a critical determinant of the quality and efficiency of health care [5], and with this goal in mind three high profile national reports called for an urgent expansion of capability and capacity of the imaging support workforce [6–8]. These reports support the planned implementation of sustainable mechanisms to improve and scale support worker utilisation, but it was unclear how imaging departments were deploying the support workforce. In a 2024 scoping review of imaging support workforce roles [3], only one paper was identified as having direct relevance to support workers (pay bands 2 and 3) and there was very limited evidence of capacity generation particularly in assistant practitioner roles (pay band 4).

Further research focused directly to the deployment and utilisation of the imaging support workforce has evidenced the very limited focus on job satisfaction and career aspiration, with confusion over scopes of practice, deployment models and supervision [9–11]. Some innovative examples of positive outcomes resulting from increased support worker autonomy have raised the profile of the unregistered workforce but perhaps more significantly have indicated ways to build resilience across services [12]. The substitution of some registered radiographer roles with assistant practitioners has also been shown to offer a cheaper workforce, and in some cases slightly higher throughput and faster examination times for patients [13]. These studies collectively highlight the importance of developing opportunities for support workforce roles as a continuum across the radiography career pathway [9–13], in part because of the serious shortfall in training capacity through traditional routes and a lack of strategic workforce planning [14, 15]. This has led to further impetus for leaders to plan for a more sustainable workforce with a greater skill mix.

This paper reports on the final stage of a multi-centre mixed-methods investigation [16] to highlight critical determinants for deployment of an effective imaging support workforce. Critical determinants are causal factors which control or influence the likelihood of something happening, such as a service running effectively [17] or unregistered personnel being properly deployed in a service. The aim was to illustrate the determinants within a maturity matrix to enable a visual and accessible representation of the key findings to support unbiased, evidence-based review by healthcare managers.

A maturity matrix is a model for assessing and improving the maturity of health care practices, operations and infrastructure [18]. It is a recognised way of translating research findings into an actionable tool that can be used by organisational management as “a reference framework that defines different levels of proficiency or effectiveness in a specific domain” (p1) [19]. Empirical benefits of using maturity matrices include improving efficiency, effectiveness, performance, and productivity [18]. Maturity matrices are being implemented with increasing frequency in healthcare, with some of the first health care maturity matrices being introduced in primary medical care [20] and organisational development in general practice [21]. In workforce domains, successful implementation can be seen with NHS England’s advanced practice maturity matrix [22] that seeks to ensure staff are working creatively across their scope of practice but within appropriate governance infrastructure. Imaging service managers are likely to be familiar with maturity matrices as they have been deployed to support implementation of several workforce initiatives, including the recent establishment of Imaging Networks in 2021 [23]. No such tool to guide managers in support workforce planning exists in imaging, nor indeed within wider healthcare settings.

The Imaging Support Workforce Maturity Matrix is presented for review as an actionable tool in the goal to build resilience and to sustain the diagnostic imaging workforce. This paper therefore addresses a critical challenge within radiography professional practice and services design, that being the full and extended deployment, progression and planning for the support workforce.

## Method

**Fig. 1:**
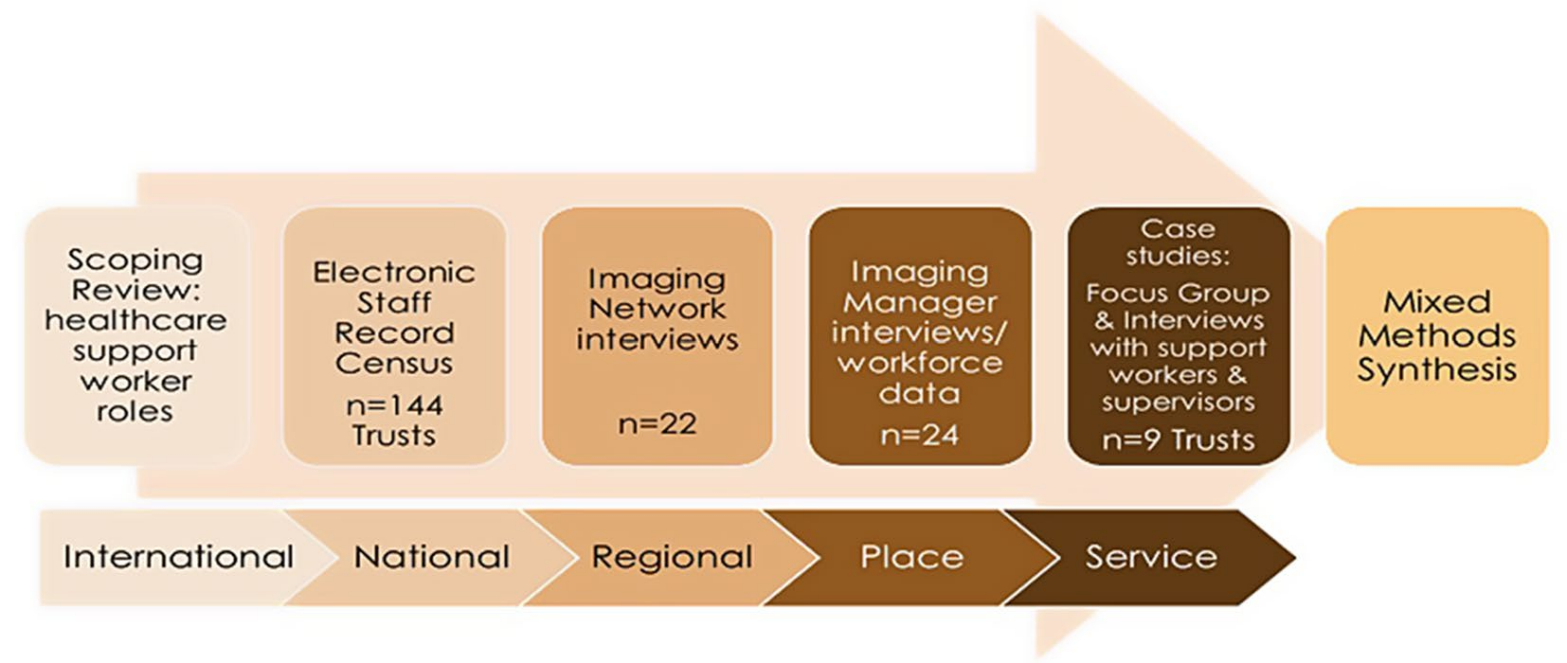
Mixed methods explanatory sequential design, with six workstreams moving from international level analysis through to service level case studies

The mixed methods study *‘The determinants of the utilisation of the support and assistant workforce in diagnostic imaging: a multi-methods investigation’* (NIHR133813) [16] known as ‘I-SWAP’ employed an explanatory sequential, multi-stage advanced framework design, involving six consecutive workstreams (Fig. 1). All required national and institutional ethical approvals were obtained [Health Research Authority 22/HRA/4272; Sheffield Hallam University Research Ethics Committee ER53139410 and ER50766713], alongside Health Education England gatekeeper permission to access anonymised data from the NHS Electronic Staff Record. Informed consent to participate was obtained from all participants in the study.

The final stage of the study, presented here, included a cross cutting mixed methods synthesis of the previous workstream outcomes, integrating the findings using a triangulation protocol method [24] alongside the O’Cathain et al. (2010) mixed methods matrix approach [25]. This method enables additional understanding where findings from each of the workstreams agree (convergence), offer complementary information (complementarity), or contradict each other (discrepancy or dissonance) [25]. The approach also enabled the visual representation of headline findings and a categorisation of the results within a matrix that tabulates the criteria against prescribed standards.

The headline findings from each stage were reviewed by the lead investigator working alongside a health policy researcher, who had no prior involvement in the previous workstreams, to reduce bias and increase trustworthiness. Key critical determinants and characteristics relating to the deployment and progression of the imaging support workforce, as indicated in previous work packages, were developed into a determinant framework [26]. The categorisation of determinants specified factors that acted as barriers and enablers (independent variables) that affected implementation outcomes (dependent variables). The framework was then populated with published results from the previous five workstreams [2, 3, 9–11], identifying ‘levels of effectiveness’ associated with each determinant.

The determinant framework was expanded into a maturity matrix, with each determinant categorised across four levels of effectiveness, using terms that would resonate with the intended professional user-group (emerging, developing, maturing, thriving). Healthcare maturity models such as the Imaging Networks matrix [23] commonly use five levels, though models with three, four or six levels also exist, such as the four level Advanced Practice matrix [22]. A higher number of options provides greater granularity but can be more complex to use, particularly to those unfamiliar with using maturity matrices. Four levels were selected as a suitable compromise, with each critical determinant carefully mapped against the four levels to present a series of distinct, iterative steps that show a desired developmental path towards excellence or efficacy. The aim was to enable workforce leaders to self-assess their current support workforce deployment against the maturity matrix, and understand, explain and predict influences on the effective implementation of support workforce interventions. An action plan would be created to address individual determinants where service improvements could be implemented.

The use of critical realism as a flexible deductive process of data synthesis and presentation [27] underpins the development of the maturity matrix and is consistent with the ambitions for the research to drive improvement in workforce planning nationally. Critical realism embraces data interpretation and methodological pluralism [28] for the purpose of creating a tool that can be used in enabling systems changes [29]. This approach recognises that variations in context and service configuration (size, history and structure) may alter the success factors for purposeful action, which in this case related to the active deployment of the imaging workforce. A maturity matrix seeks to bring together these complex factors that are necessarily incorporated into planning decisions allowing complexity to ‘play out’ [30]. The maturity matrix acts as a rubric [31] for self-evaluation of a current situation, helping managers to make a more systematic evaluation of the circumstances in a particular setting.

The first draft of the maturity matrix was reviewed by the research team as an initial validation exercise. The second draft was shared with a Stakeholder Advisory Group of imaging managers and support workforce representatives for applicability to imaging services within the wider NHS. The final draft was reviewed at a national stakeholder event which included representatives of imaging services, imaging networks, integrated care systems, policy makers and the professional body (Society and College of Radiographers, SCoR). The definitive version, alongside a spreadsheet to facilitate action planning, was subsequently shared with senior representatives of NHS England and the SCoR.

### Findings

Review of the published findings of the previous workstreams [2, 3, 9–11] established unequivocally that the support workforce is highly valued by imaging networks, imaging managers, modality leads and radiographer supervisors. Four significant findings were identified:

1. Operational efficiency and service impact, where the support workforce was critical in optimising workflows.

2. Roles and responsibilities, recognising both role clarity and ambiguity leading to role strain.

3. Career progression, support, and training, highlighting opportunities yet significant barriers to advancement.

4. Workforce dynamics and job satisfaction, where high job satisfaction contrasted with challenges in role stability and professional recognition.

These findings highlighted wide and unwarranted variations in the deployment of the support workforce, including role grading discrepancies, a preference for rotational or static deployment models, and a lack of innovation in many roles, including under-utilisation of assistant practitioners. The research has clearly identified that the imaging support workforce in England is operationally managed rather than strategically planned [10]. In the absence of clear published guidance, most services undergo development in isolation, exposing the imaging support workforce to local variation in terms of deployment models, role visibility and development opportunities.

**Fig. 2:**
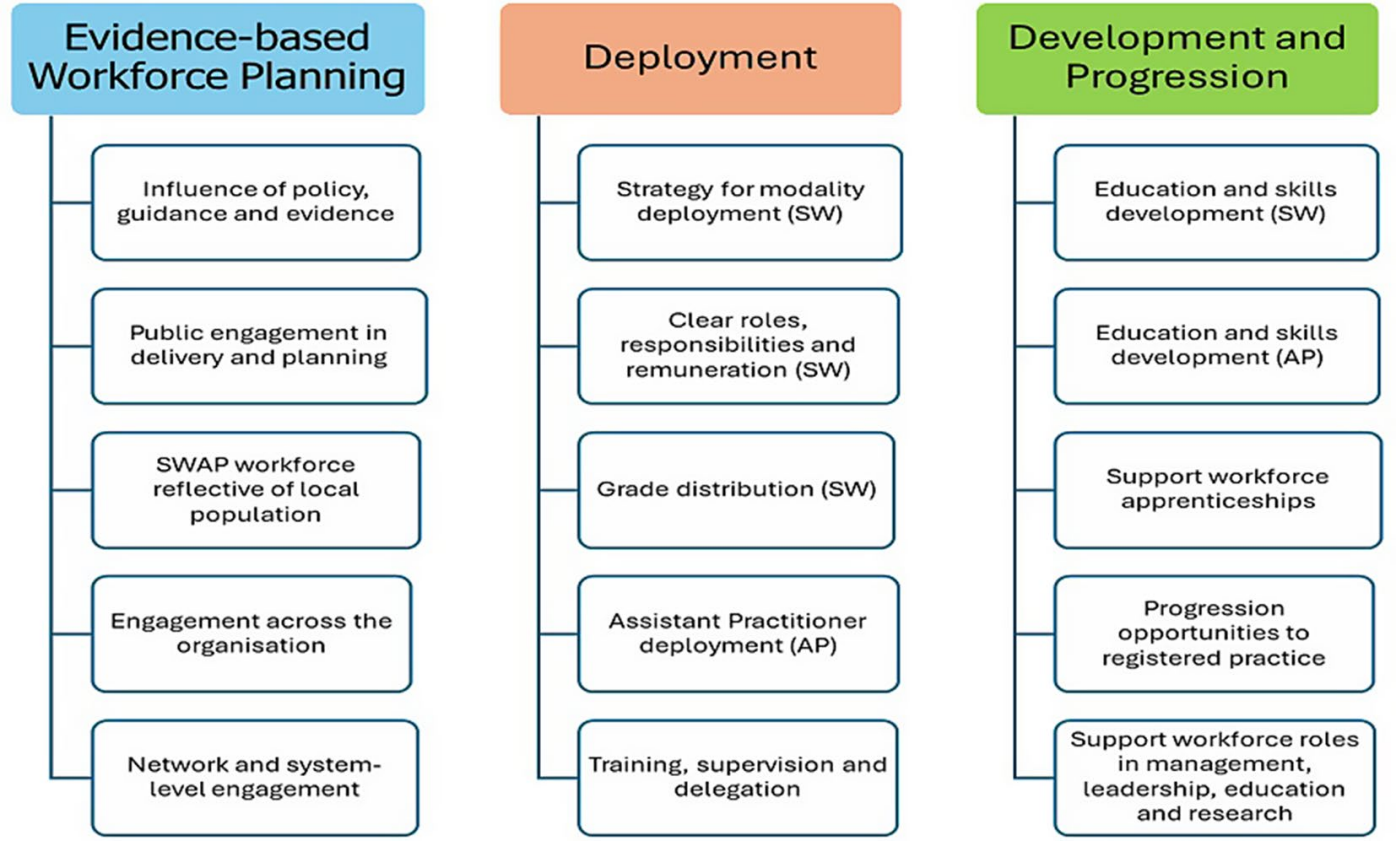
Three themes and fifteen critical determinants of the deployment of the support workforce arising from the mixed methods synthesis. adapted from Nightingale et al^16^

The Imaging Support Workforce Maturity Matrix was designed to support services to strategically review their support workforce and, where relevant, to compare and contrast with other imaging services in their imaging network. The matrix comprises three over-arching themes recognised in this research as pivotal to the effective use of the workforce: Evidence-based Workforce Planning; Deployment; Development and Progression. The three themes each encompass five critical determinants for the effective deployment and utilisation of the imaging support workforce (Fig. 2).

The three matrix themes and their critical determinants are outlined in Tables 1, 2 and 3. The Evidence-Based Workforce Planning theme (Table 1) encourages workforce leaders to engage in strategic review of their workforce both within and across organisations, underpinned by relevant evidence and policies, in addition to public involvement and engagement. This theme is informed by findings from interviews with imaging service and network leaders [10] which identified a widespread lack of engagement with, or knowledge of national policy and guidance. Importantly, few imaging leaders had reviewed the diversity of their support workforce and they were unaware whether it was reflective of the local population. No participants had engaged with public groups in their workforce planning activities, and few had engaged with support workforce leads either within or external to their organisation. Regional Imaging Networks were highlighted as a future opportunity for sharing best practice and developing collaborative workforce plans [10].

The Deployment theme (Table 2) supports workforce leaders to critically review and justify their deployment strategies, and ensure safe, transparent and explicit frameworks for roles, responsibilities and supervision. This theme is informed by the Electronic Staff Record census [11] which demonstrated unwarranted variations in grade utilisation, with some deploying predominantly band 2 (support workers) and some predominantly band 3 (senior support workers). Service lead interviews [10] confirmed a lack of consistency in support worker role titles, responsibilities and deployment models (rotational or single area of practice). Under-utilisation and lack of innovative roles for assistant practitioners was starkly confirmed in both the national census [11] and service lead interviews [10]. While some good practice was identified in terms of supervision and delegation [9, 10], lack of clarity often led to either under-utilisation or role creep, whereby experienced support workers gradually strayed outside their role boundaries [9].

The Development and Progression theme (Table 3) facilitates workforce leaders to review training and education opportunities through induction, preceptorship, promotion and ultimately (for some support workers), through to registered practice. In addition, managers are encouraged to consider support workforce roles beyond the clinical domain, encompassing supporting roles in leadership and management, education and research. This theme is informed predominantly by the nine case studies and subsequent cross-case analysis [9] which confirmed highly variable levels of training and development for the support workforce, though some had introduced transparent and equitable capability frameworks. While most training was ‘in-house’, some services had accessed external courses and apprenticeships, though few opportunities for support workers to progress to registered practice were available [9]. To maintain motivation and capitalise on experience and expertise, some managers had introduced innovative roles including support worker leadership opportunities, which released managers and radiographers from time-consuming duties and provided an alternative to clinical progression [9, 10]. This was seen to improve support workforce ownership, visibility and belonging.

Within each table, a red-amber-green (RAG) rating scale was added to allow the matrix-users to rate their service for each of the individual determinants, with “Emerging”, “Developing”, and “Maturing” used as labels for the red, amber, and green levels, and then a final blue level labelled “Thriving” for the highest rating. Each of the critical determinants (column 1) are closely aligned with the outcomes and data related to the previously published findings, with quotes or examples from the data enabling explanation or animating the meaning of the issue. Each critical determinant is supplemented by a rationale for the importance of the issue in column 2.

The Imaging Support Workforce Maturity Matrix document was embedded within an interactive online spreadsheet to enable managers to engage with the content meaningfully. This spreadsheet encouraged initial review of each determinant alongside detailed action planning, supporting periodic review of maturity ratings. The online tool can be accessed on the project website [32] and in Supplementary Materials.

**Table 1:** Workforce theme 1: evidence based workforce planning

Determinant	Importance	Emerging (E)	Developing (D)	Maturing (M)	Thriving (T)	Level (RAG-B)
1.1 Support workforce plans underpinned by national policy, guidance and research evidence	Engagement with published research, guidance and frameworks supports best practice and reduces unwarranted variation, providing evidence for business planning and improving support workforce morale and transferability between Trusts	No/limited awareness of current professional or policy publications related to the support workforce	Working knowledge of professional support workforce resources, e.g. SCoR/HEE guidance on roles and responsibilities and supervision	Understanding of professional support workforce guidance and cross-discipline workforce resources, e.g. HEE AHP Support Worker Competency Framework	Benchmarking against relevant policy and tools (e.g. Model Hospital). Guidance has informed workforce planning and scope of practice review.	
1.2 Public engagement in support workforce service delivery and planning	Engagement with Patient and Public Involvement (PPI) groups (e.g. Patient Advice and Liaison Services – PALS) can offer a different perspective on support workforce priorities and patient engagement across pathways which may be useful in business planning	No public or PALS engagement or mechanism for public awareness of support workforce in imaging service delivery	The role and contribution of support workforce to imaging service delivery promoted (alongside the registered staff) to patients and the public. Initial engagement with PALS.	Understanding of local context and population (informed by PALS) included in support workforce planning, for example opportunity for roles focussed on specific population needs.	Population demographics /context is known (data) and used in planning with regular mechanisms for PPI/PALs engagement (e.g. patient advisory panel, Experts by Experience)	
1.3 SWAP workforce reflective of local population	SWAP workforce reflects local population and supports diversity in the wider imaging workforce. Creates role models in service and wider community, improving recruitment, retention, opportunity and staff morale.	Limited or no understanding of workforce drivers related to Equality, Diversity, Inclusion and Belonging (EDIB)	Initial engagement with community or cultural leaders. Some understanding of significant local EDIB challenges but no action planning	Review of service SWAP workforce profile (age, gender, ethnicity) to include EDIB review of recruitment and progression	Strategic review of staff workforce profile informing recruitment strategies to enable the SWAP workforce to reflect diverse local population	
1.4 Engagement across the organisation	Engaging in support workforce developments across the organisation increases visibility, enabling inclusion of imaging support workforce in relevant multi-disciplinary networks, training, and progression	No strategic involvement or awareness of Trust support workforce networks	Representation on relevant Trust groups relevant to the support workforce including education and planning	Discussions in Trust networks and groups inclusive of imaging support workforce with relevant resources identified	Influence at Trust level including support workforce training, apprenticeships, workforce planning and progression. Engagement at network or system level is evident.	
1.5 Network and System Level Engagement	Network and/or system level engagement highlights innovative support workforce practices and provides opportunities to reduce variance and capitalise on new ways of working, whilst improving clarity of roles and the potential for sharing training and other resources	Support workforce is not included in any regional network or system imaging or AHP discussions	Regional networks focusing on the AHP or imaging-specific support workforce but no review of variations in imaging scope of practice or deployment	Regional scoping of the imaging support workforce deployment, roles, scope of practice and agreement to share learning	Imaging managers engaging with support workforce strategic planning activities in Imaging Networks or AHP Faculties	

**Table 2:** Workforce theme 2: deployment

Determinant	Importance	Emerging (E)	Developing (D)	Maturing (M)	Thriving (T)	Level (RAG-B)
2.1 Strategy for support worker (SW) modality deployment (Bands 2/3)	Balance of flexibility (rotation) and skills development (modality deployment) to enhance SW satisfaction, promote team building, and enhance patient pathways and experience	Custom and practice (static / rotational) not questioned	SW deployment models reviewed, and action plan developed. Support workforce largely rotational with emerging static posts, enabling skills development, team working and contribution	SW deployment models responsive to imaging service need. Where appropriate flexible rotational posts to enable cross-modality ‘cover’.	SW deployment strategy embedded - rotation used within induction for familiarisation, specialist deployment used for SW skills enhancement	
2.2 Clear roles, responsibilities and remuneration (Bands 2/3)	Consistent support worker identity across services, organisations and networks increases visibility and improves recruitment, retention and progression opportunities whilst offering opportunities for cross system working	No consistency in support worker role titles and grades across the imaging service(s)	Some role titles are consistent, but grades and role responsibilities vary across the imaging service(s)	Clearly visible roles for support workers via consistent job titles aligned to grade and wider organisational appointments	Support worker identity, roles, titles, grades consistent across services and imaging networks. Opportunities for system working explored and/or implemented	
2.3 Support worker (SW) grade distribution (Bands 2/3)	Grade balance is strategically planned and related to SW responsibilities rather than relying on custom and practice, enabling support workforce training and educational opportunities, progression and improved morale. Aligned to employing organisation policies and procedures.	No current or recent strategic focus or review of grade distribution (Band 2 and 3) across the service	Localised decisions about grading with reference to context and local pressures for staffing recruitment and retention	Local review of workforce structures to clearly distinguish Band 2 from Band 3 within and across services with reference to career progression and recruitment pressures	Clear accountability in service / across networks for role delivery and balance of support worker staffing to enable recruitment, retention, skills mix and progression	
2.4 Assistant Practitioners (AP) (Band 4)	Effective deployment of Assistant Practitioners supports innovations in patient care and delivery. It supports radiography skills mix and support worker progression and retention. APs may offer an additional employment pool for pathway to registered practice (local workforce with improved retention)	Not utilising APs	Band 4 AP roles used in limited modalities or deployed as training roles within Apprenticeships. Governance and scope of practice review required.	Review of potential deployment opportunities with plan for engagement of wider AP roles. Opportunities explored for radiographer/AP skill mix review across modalities.	AP roles embedded and potential fully realised. Governance in place aligned to defined scope of practice. Providing or considering progression opportunities (e.g. Band 5 Associate Practitioner).	
2.5 Training, supervision and delegation	Support workforce roles can only take place in the presence of clear supervisory and delegation policies. Both registered and support staff require a clear understanding, with training offered to new employees	Lack of clarity in roles and responsibilities, no training specific to supervision or delegation provided	Active engagement with support workforce and registered staff to review role supervision and delegation requirements	Clear supervision policy with associated training offer for current and new support workforce and registered staff	Clear understanding of scope of practice and supervision requirements enables support workforce innovations	

**Table 3:** Workforce theme 3: development and progression

Determinant	Importance	Emerging (E)	Developing (D)	Maturing (M)	Thriving (T)	Level (RAG-B)
3.1 Education and skills development for Support Workers (Bands 2 and 3)	Rolling education and training programme ensures a competent support workforce and a clear career trajectory into senior support workforce roles. Improves staff morale, recruitment and retention. Training of registered staff ensures safe and effective delegation and supervision.	Requirement for role specific education and training recognised. Training needs analysis considered against role requirements.	Competency frameworks in place for support staff across modalities but no underpinning resources or training plans.	Training packages for initial education to meet competency frameworks. Ongoing provision of CPD for support workers in place, guided by SCoR Education and Career Framework.	Service changes consider support staff education. New skill acquisition underpins progression. Peer mentor and education roles embedded. Liaison with FE Colleges evident	
3.2 Education and skills development for Assistant Practitioners (Band 4)	An education and training offer designed for the Assistant Practitioner (AP) workforce supports a fully utilised scope of practice guided by SCoR Education and Career Framework and provides clear career opportunities improving recruitment, retention and staff morale.	Department-based training reflects the scope of practice for the role, but academic underpinning is limited or does not enable progression	AP roles supported by formal recognised education programmes, but not enabling progression	CPD opportunities include APs either alongside registered staff or with specific role focussed training. Organisational level engagement with education provider(s).	Education supports skills maintenance / expansion, enabling progression. Education provider(s) engaged at service level, influencing content and future provision.	
3.3 Support Workforce Apprenticeships	Rolling programme of support workforce apprenticeships (Academic Levels 2/3,5) provides regular progression opportunities to support recruitment and retention and improve workforce morale. Developments underpinned by SCoR Education and Career Framework.	No opportunities for apprenticeships (Level 2/3/5) and no opportunities for access to training beyond mandatory courses.	No access to Level 2/3/5 apprenticeships, other formal training available. Access to functional skills (Maths/English) through organisation.	Opportunity for apprenticeships at one or more academic levels, though number of places limited restricting progression opportunities.	Apprenticeships across academic levels available on a rolling programme across support workforce levels. Clear succession planning.	
3.4 Progression opportunities to registered practice	A rolling programme of AP ‘top up’ courses and/or Degree Apprenticeships (DA) to support recruitment into radiography posts increases retention and widens diversity (local workforce)	Not planning to offer DA or top up opportunities or other progression to registered practice	Not yet offering DA/top up options, exploring options with apprenticeship leads and HEIs and preparing business case	Small annual DA or top up intakes and/or supporting SWAPs to progress to traditional routes (e.g. Top up degrees).	DA or top up on a rolling programme, functional skills offer to ensure wide and inclusive learning opportunities	
3.5 Engagement beyond clinical roles: Leadership, Management, Research and Education	Support workforce engagement in innovative roles may release managers and radiographers from time-consuming duties, providing an alternative to clinical progression. Improves support workforce ownership, visibility and belonging.	No engagement evident beyond clinical practice. Management and Leadership solely from registered workforce.	Limited engagement in peer education. Some opportunities for enhancing SWAP voice or participation	Support Workforce engages in peer mentorship, training and management roles (e.g. rostering), with tasks appropriately delegated	Wide engagement beyond clinical roles; support workforce leadership representation in imaging service decision-making forums	

## Discussion

The imaging workforce is widely regarded as ‘in crisis’ with the demand for imaging outstripping the available service capacity [33]. Persistently high vacancy rates for both radiologists and radiographers [34–36], continue to impact on both patient waiting lists for imaging and on imaging report turnaround times [34]. In this context the radiography profession is being encouraged to use the support workforce in more innovative ways, developing their capacity and capability so that their full potential can be realised [5, 7, 8]. Maximising efficiency in the support workforce requires a clear vision and strategic action planning to work towards the most effective mix of grades (bands 2–4), deployment model (rotation versus specialist) and generation and adoption of innovative roles.

The Imaging Support Workforce Maturity Matrix offers the first interactive tool for managers to systematically evaluate the factors associated with the best use of their support workforce. The ‘Matrix’ is based on research evidence that enables managers to better understand their context and to evaluate factors for improvement, for example, how to effectively train, develop, and deploy their imaging support workforce. It is designed to encourage discussion, debate, benchmarking and action planning both within and across imaging teams and networks. To our knowledge, the collation of evidence as an actionable tool is a new initiative within the Allied Health Professions (AHP), facilitated by the outputs of the ‘I-SWAP’ research programme [16].

The matrix complements UK professional and AHP support workforce frameworks and guidance documents [37–40], providing an evidence-base for implementation and promoting attention on support worker strategies which could enable NHS Trusts to fulfil one of their roles as an ‘anchor’ institution’ [41], insofar as widening access to healthcare roles and applying equality, diversity and inclusion initiatives in local communities. This is particularly important for NHS Trusts in deprived rural, coastal, and urban areas, where communities are likely to have the greatest need, but where the hospitals experience more recruitment and retention challenges [42]. The engagement with local communities responds to the call for evidence from NHS employers associated with rural workforce planning [43].

A potential limitation of the imaging support workforce maturity matrix is that the evidence on which it is based is drawn from the National Health Service in England, so may not be directly transferable to other countries. The primary focus is imaging services, however the maturity matrix is likely to be applicable to radiotherapy services, where the support workforce are deployed in a similar department-based structure where imaging is a key component of radiotherapy planning and treatment review. It may also be applicable to other allied health professions who are equally lacking in formal workforce planning tools. Following good practice in the validation of other maturity matrix tools [21], further work is underway to measure the impact, utility and transferability of the maturity matrix.

## Conclusion

Implementation of research outcomes is best achieved via actionable tools that engage and share evidence-based practice with managers and leaders for the purpose of improving services. The Imaging Support Workforce Maturity Matrix incorporates evidence-based critical determinants into a framework to promote assessment, critical review and discussion within and across imaging workforce teams. The implementation of this matrix will enable the furtherment of support workforce roles, with improvements in staff experience and department efficiency. If successful in this, these modifications will have a subsequent effect on patient experience within imaging departments, by providing a stable and thriving workforce within an efficient and effective department.

## Supplementary Information

Below is the link to the electronic supplementary material.

Supplementary Material 1

## Data Availability

The datasets generated and/or analysed during the current study are available in the Sheffield Hallam University Research Data Archive repository, [https://shurda.shu.ac.uk/] upon reasonable request to the corresponding author. The Imaging Support Workforce Maturity Matrix tool and interactive action planning spreadsheet is available at [https://research.shu.ac.uk/i-swap/?page_id=238].

## Abbreviations

AHP: Allied Health Professions
NHS: National Health Service
SWAP: Support Worker and Assistant Practitioner
UK: United Kingdom
